# *Pneumocystis jirovecii pneumonia* in HIV-uninfected, rituximab treated non-Hodgkin lymphoma patients

**DOI:** 10.1038/s41598-018-26743-4

**Published:** 2018-05-29

**Authors:** Kai-Che Wei, Chenglen Sy, Shang-Yin Wu, Tzu-Jung Chuang, Wei-Chun Huang, Ping-Chin Lai

**Affiliations:** 1 Department of Dermatology, Kaohsiung Veterans General Hospital, Kaohsiung, Taiwan; 2 Division of Infectious Diseases, Department of Internal Medicine, Kaohsiung Veterans General Hospital, Kaohsiung, Taiwan; 3 Division of Hematology/Oncology, Department of Internal Medicine, National Cheng Kung University Hospital, College of Medicine, National Cheng Kung University, Tainan, Taiwan; 4 Critical care center and Cardiovascular Medical Center, Kaohsiung Veterans General Hospital, Kaohsiung, Taiwan; 5 School of Medicine, National Yang-Ming University, Taipei, Taiwan; 6 Transplantation laboratory, Department of Nephrology, Kidney Center, Chang Gung Memorial Hospital, Chang Gung School of Medicine, Chang Gung University, Linkou, Taiwan; 7 China Medical University Hospital, China Medical University, Taichung, Taiwan

## Abstract

Rituximab is associated with a higher incidence of *Pneumocystis jirovecii pneumonia* infection. Pneumocystis prophylaxis is advised in many immunocompromised populations treated with rituximab. However, the beneficial effect of pneumocystis prophylaxis in HIV-uninfected, rituximab-treated non-Hodgkin lymphoma (NHL) patients has not been assessed. Thus, we conducted this retrospective study to explore pneumocystis infection in HIV-uninfected NHL patients who received at least three courses of chemotherapy without haematopoietic stem cell transplantation using the Taiwan National Health Insurance Research Database. Patients who had rituximab-based chemotherapy were included in the experimental (rituximab) group, while the rest of the patients who did not receive any rituximab-based chemotherapy throughout the study period formed the control group. The prevalence rate of pneumocystis infection in the rituximab group (N = 7,554) was significantly higher than that in the control group (N = 4,604) (2.95% vs. 1.32%). The onset of pneumocystis infection occurred between 6 and 16 weeks after chemotherapy. Patients who had pneumocystis prophylaxis, whether or not they had a pneumocystis infection later in their treatment course, had significantly better first-year survival rates (73% vs. 38%). Regular pneumocystis prophylaxis should be considered in this group of patients.

## Introduction

*Pneumocystis jiroveci* pneumonia, formerly known as *Pneumocystis carinii* pneumonia (PcP), occurs when immune function is suppressed to a certain threshold. Noticeably, once these immunocompromised patients are infected, the mortality rate can be as high as 30 ~ 60%^1,2^. To make things worse, a delay in diagnosis is not uncommon because its initial manifestations are usually nonspecific and include fever, dry cough and pulmonary interstitial infiltrates.

Rituximab is a monoclonal antibody that binds to the CD20 antigens on B lymphocytes and leads to B cell elimination from the body. Through this effect, rituximab has been proven effective in many B cell-associated diseases, such as B cell lymphoma and inflammatory autoimmune disorders. The widespread application of rituximab was soon followed by reports linking pneumocystis infection to rituximab^3–5^. Thanks to previous studies in HIV/AIDS patients, it is understood that pneumocystis infection is mostly associated with T cell dysfunction. Therefore, when pneumocystis infection occurs in rituximab-treated patients, rituximab, an anti-B cell agent, appears innocent at first glance. However, recent evidence demonstrates that B cell dysfunction can also lead to pneumocystis infection. In a murine study, Elsegeiny *et al*. elegantly showed that anti-CD20 antibody alone was permissive to pneumocystis infection by impairing type II immune responses and causing CD4+ T cell dysfunction in lung tissue^6^. In another study, Lund F. E. *et al*. showed that both CD40 and B cells are critical for CD4+ T-cell activation and thus for defence against *Pneumocystis jirovecii* infection^7^. Similar findings were found in a human peripheral blood study^8^. In addition, rituximab can cause prolonged hypogammaglobulinemia and hinder naive B lymphocyte differentiation into plasma cells, which are crucial for eliminating *Pneumocystis jirovecii*^6,9^. Together, these studies showed convincingly that the integrity of B cell function is critical for immune reactions against pneumocystis infection. Thus, it is not surprising that the prevalence rate of pneumocystis infection is higher among patients receiving rituximab-based chemotherapy than among those receiving rituximab-sparing regimens^4,5,10–12^.

Cytomegalovirus (CMV) infection is a T cell-related opportunistic infection. Several studies have shown that it occurs frequently among haematology patients. Moreover, co-infection with other microbes is not uncommon^13,14^. Thus, CMV infection is another infection that may be found in immunocompromised patients. Therefore, we decided to compare CMV to pneumocystis infection to determine if pneumocystis infection is just a complication reflecting a general immunocompromised state or if it actually represents a specific risk to rituximab-treated patients.

Although life threatening, pneumocystis infection is preventable by the administration of oral trimethoprim/sulfamethoxazole (TMP/SMX). Thus, when rituximab is administered for various diseases, regular pneumocystis prophylaxis with TMP/SMX is widely recommended by many treatment guidelines, such as Wegener’s granulomatosis and organ or haematopoietic transplant^15,16^. However, the necessity of pneumocystis prophylaxis for HIV-uninfected, rituximab-treated non-Hodgkin lymphoma (NHL) patients remains controversial because the benefits and risks of prophylaxis have not been assessed in a large-scale study^4,10,17^. Thus, in this report, we investigated the beneficial effect of pneumocystis prophylaxis with oral TMP/SMX in HIV-uninfected NHL patients by analysing data from a national registry, the Taiwan National Health Insurance Research Database (NHIRD). The results of this study will provide further evidence to clarify if pneumocystis prophylaxis is beneficial to HIV-uninfected, rituximab-treated NHL patients.

## Materials and Methods

In Taiwan, National Health Insurance has covered over 99.9% of the 23 million Taiwanese people since 1995. To respond rapidly and effectively to the current and emerging health issues, the National Health Research Institute (NHRI) established the Nation Health Insurance Research Database (NHIRD). The NHRI safeguards the privacy and confidentiality of each patient and routinely updates this database.

The study was designed according to the principles of the Declaration of Helsinki and was approved by the IRB of Kaohsiung Veterans General Hospital (VGHKS16-CT11–08 and VGHKS15-EM10-02). The IRB exempted the requirement for written informed consent because the NHIRD is a de-identified and encrypted database. The main files used in this study included the catastrophic illness dataset (HV file) and the NHIRD from January 2006 to December 2013. The HV file is a national registry for catastrophic illness. To be listed in this registry, medical records of all the applicants have to go through a formal review protocol held by the National Health Insurance Administration committee for an exempted copayment. All the data retrieved from the NHIRD were reconfirmed using data from the HV file.

The primary objective of this retrospective cohort study was to investigate if trimethoprim/sulfamethoxazole prophylaxis is beneficial to the HIV-uninfected, rituximab treated NHL patients. To answer this question, we first defined the prevalence rate of pneumocystis infection in these patients and then clarified if pneumocystis prophylaxis with trimethoprim/sulfamethoxazole is beneficial to overall first-year survival.

**Figure 1:**
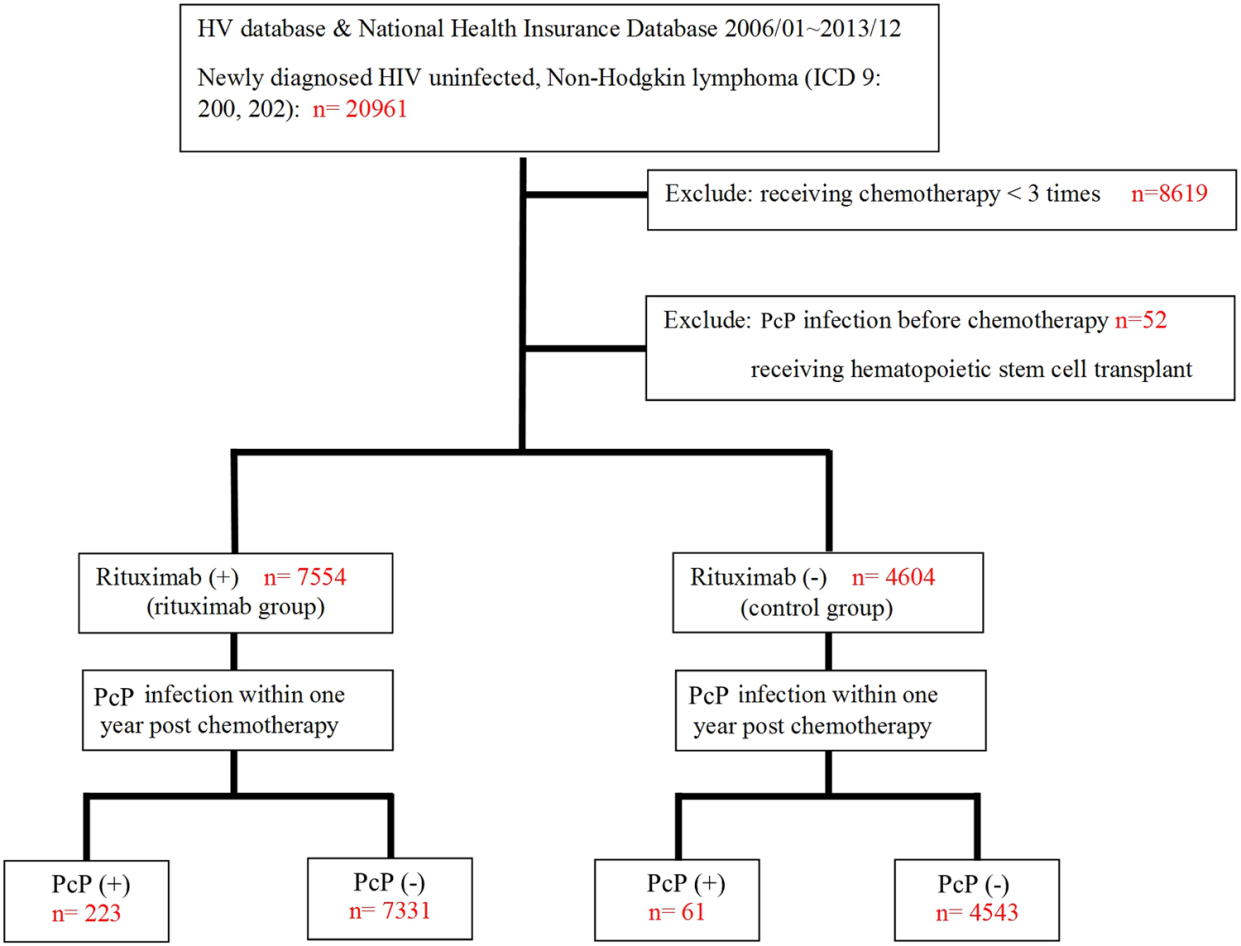
The study flow of *Pneumocystis jirovecii pneumonia* infection (PJP, formerly known as PcP) in HIV-uninfected non-Hodgkin lymphoma patients from Jan/2006 to Dec/2013.

The details of the study flow and plan are presented in Fig. 1. We included patients with HIV-uninfected NHL, which was defined by the International Classification of Disease, 9th Revision, Clinical Modification (ICD-9-CM) codes for NHL (200.0–200.8 and 202ν–202.9). Data for these patients were then validated by using data from the catastrophic illness dataset (HV file) to assure the accuracy of this study. Once included, demographic data, comorbidities, chemotherapy regimens, and first-year survival rate were collected and analysed. The patients who had rituximab-based chemotherapy were included in the experimental group, while the rest of the patients who do not receive any rituximab-based chemotherapy throughout the study period formed the control group. The major goal of the study was to compare the prevalence rate of pneumocystis infection between the experimental (rituximab) group and the control group during the first year post-chemotherapy and the beneficial effect of trimethoprim/sulfamethoxazole (TMP/SMX) prophylaxis. To exclude the disease progression effect, only patients who received at least three courses of chemotherapy were included in this study. Patients who were infected with HIV, had Hodgkin lymphoma or received haematopoietic stem cell transplantation were excluded from this study.

The pneumocystis-infected cases were identified by codes for pneumocystis infection (136.3), which had to be recorded during admission, and by the use of intravenous trimethoprim/sulfamethoxazole (TMP/SMX). Patients who had a pneumocystis infection before the initiation of chemotherapy were excluded. The prescription of oral TMP/SMX at the outpatient clinic prior to the diagnosis of pneumocystis infection was defined as primary prophylaxis. Cases with another opportunistic infection, cytomegalovirus (CMV) infection, were identified by codes for CMV infection (078.5).

Although the NHIRD has the advantage of nation-wide coverage and a large number of patients, its drawback is the lack of medical details for each patient. To assess the impact of this weakness and to validate the reliability of this study, we conducted a pilot study with the same inclusion/exclusion criteria plus a detailed medical record review in our hospital (supplement 1).

## Statistics

The frequencies of each categorical variable were compared with chi-square (χ^2^) tests and/or Fisher’s exact tests. Propensity score matching was performed to correct for sample selection bias. Kaplan-Meier analyses were used to calculate the survival rate of the pneumocystis-infected and pneumocystis-free groups, and log-rank tests were used to compare the survival differences between these two groups of patients. A multivariate analysis of clinical variables associated with the diseases was performed with Cox regression.

## Results

By examining the NHIRD, we identified 20,961 HIV-uninfected NHL patients from January 2006 to December 2013. After excluding patients who were ineligible for this study, we found 7554 HIV-uninfected NHL patients who received rituximab-based chemotherapy (rituximab group) and 4604 HIV-uninfected NHL patients received chemotherapy without rituximab (control group). The result showed that the prevalence rate of pneumocystis infection was significantly higher in the rituximab group than in the control group (2.95% vs. 1.32%; p < 0.001) (Table 1). This result was validated by a smaller single-centre study done with the same inclusion/exclusion criteria and included a detailed medical record review of each patient (supplement 1). Importantly, there was no difference in the prevalence rate of another T cell-related opportunistic infection, CMV infection, between these two groups of patients (0.99% vs. 0.98%; p = 0.9334) (Table 1), indicating that pneumocystis infection is a unique risk to this group of patients.

**Table 1:** Demographic data and prevalence rates of *Pneumocystis jirovecii pneumonia* (formerly known as *Pneumocystis carinii pneumonia* (PcP)) and CMV infection in HIV-uninfected non-Hodgkin lymphoma patients.

	Rituximab group N = 7554 (100%)	Control group N = 4604 (100%)	*p* value
Age (y)	61.0 ± 16.0	49.5 ± 21.5	<0.0001
Gender ratio			<0.0001
Male	4138 (54.7%)	2808 (60.9%)	
Female	3416 (45.2%)	1796 (39.0%)	
Chronic pulmonary disease	2624 (34.7%)	1474 (32.0%)	<0.001
Rheumatologic diseases	1849 (24.4%)	794 (17.2%)	<0.0001
Diabetes mellitus (DM)	1694 (22.4%)	717 (15.5%)	<0.0001
DM with chronic complications	455 (6.0%)	174 (3.7%)	<0.0001
Chronic kidney disease	1849 (24.4%)	794 (17.2%)	<0.0001
*Pneumocystis jirovecii pneumonia* (PcP)	223 (2.95%)	61 (1.32%)	<0.0001
Cytomegalovirus (CMV)	75 (0.99%)	45 (0.98%)	0.93

**Figure 2:**
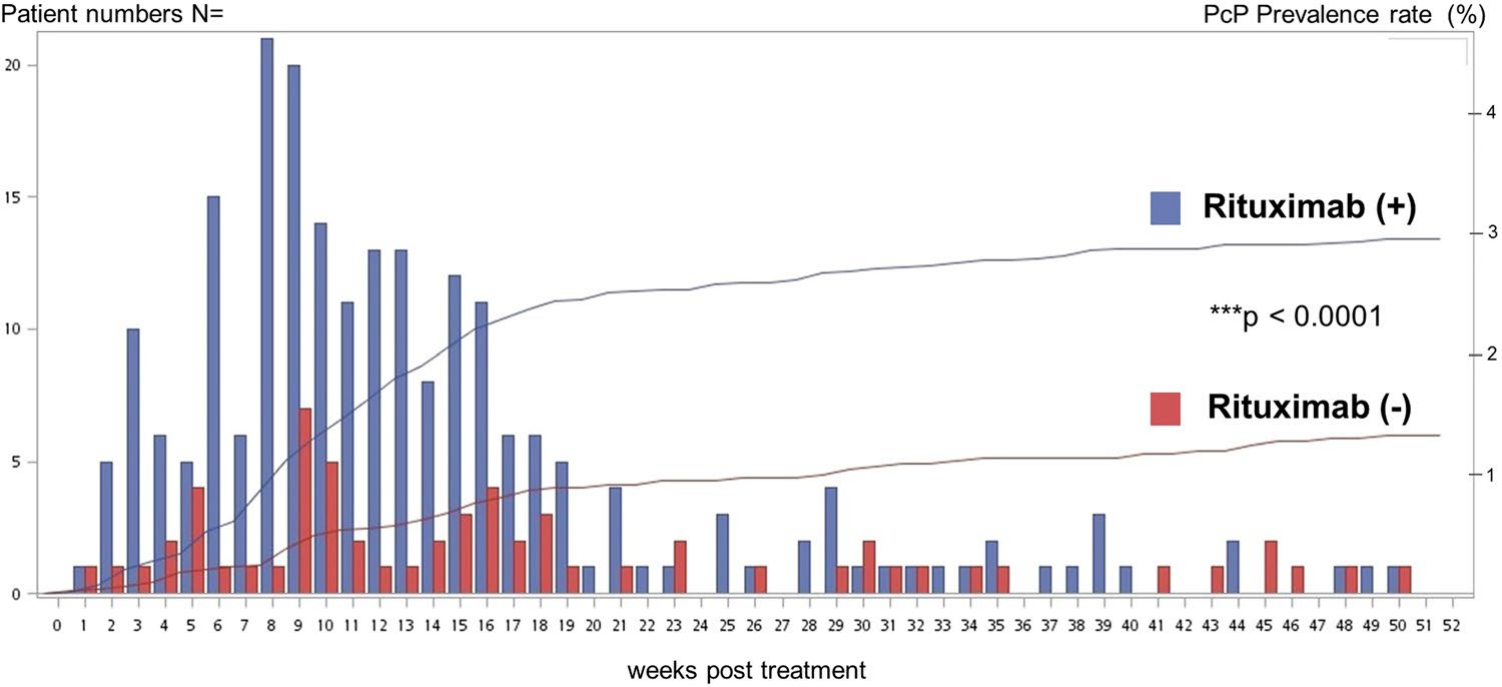
The incidence (bars) and cumulative prevalence (lines) of pneumocystis infection at different time points post chemotherapy in HIV-uninfected non-Hodgkin lymphoma patients. (blue bar/line: rituximab group, red bar/line: control group) (X axis: weeks post chemotherapy; Y axis: case number). Most pneumocystis infections occurred within 20 weeks post rituximab treatment, while the peak incidence was at approximately 6 to 16 weeks post treatment.

The patients in the rituximab group, when compared to those in the control group, were older and had significantly more comorbidities, such as chronic pulmonary disease, rheumatologic disease, diabetes mellitus and chronic kidney disease (Table 1). A further analysis within the rituximab group revealed that the pneumocystis-infected patients had a similar demographic background as the pneumocystis-free patients except for a proportion of males, which was significantly higher in the pneumocystis-infected group (P < 0.0001) (Table 2). A multivariate Cox regression was performed and demonstrated only that male gender and rituximab use were associated with a higher risk of pneumocystis infection, while age and underlying comorbidities were not related to the risk of pneumocystis infection (Table 3). Moreover, we also found that pneumocystis infection occurred mostly within 20 weeks after the initiation of rituximab treatment, and the peak incidence was at approximately 6 to 16 weeks post treatment (Fig. 2).

**Table 2:** Demographic data of pneumocystis-infected (PcP (+)) and pneumocystis-free (PcP (−)) patients in the rituximab group.

	Rituximab group		
	PcP (+) N = 223 (100%)	PcP (−) N = 7331 (100%)	*p* value
Age (y)	59.4 ± 14.7	61.1 ± 16.1	0.14
Gender ratio			<0.0001
Male	150 (67.2%)	3988 (54.4%)	
Female	73 (32.7%)	3343 (45.6%)	
Chronic pulmonary disease	67 (30.0%)	2557 (34.8%)	0.13
Rheumatologic diseases	50 (22.4%)	1799 (24.5%)	0.46
Diabetes mellitus (DM)	41 (18.3%)	1653 (22.5%)	0.14
DM with chronic complications	11 (4.9%)	444 (6.0%)	0.48
Chronic kidney disease	50 (22.4%)	1799 (24.5%)	0.46

**Table 3:** Univariate and multivariable logistic regression analyses of different underlying factors for the development of pneumocystis infection.

Risk factor	Simple regression			Multiple regression		
	HR	95% CI	*P* value	HR	95% CI	*P* value
Rituximab	2.33	(1.75–3.09)	<0.0001	2.47	(1.84–3.3)	<0.0001
Gender (Male)	1.41	(1.11–1.8)	0.0057	1.51	(1.18–1.94)	0.001
Age (y)						
45–60 vs. ≤ 45	1.11	(0.8–1.52)	0.544	0.95	(0.69–1.32)	0.7687
60–70 vs. ≤45	1.27	(0.9–1.79)	0.1726	1.07	(0.75–1.54)	0.709
>70 vs.≤45	0.98	(0.69–1.37)	0.8867	0.83	(0.57–1.22)	0.3412
COPD	0.87	(0.67–1.12)	0.2786	0.9	(0.69–1.18)	0.4413
RD	0.97	(0.73–1.3)	0.8535	1.07	(0.79–1.45)	0.6751
DM	0.88	(0.65–1.2)	0.4214	0.87	(0.63–1.19)	0.3796
CKD	0.64	(0.37–1.12)	0.1159	0.65	(0.37–1.15)	0.1357

COPD = Chronic pulmonary disease, RD = Rheumatologic disease, DM = Diabetes mellitus, CKD = Chronic kidney disease.

**Figure 3:**
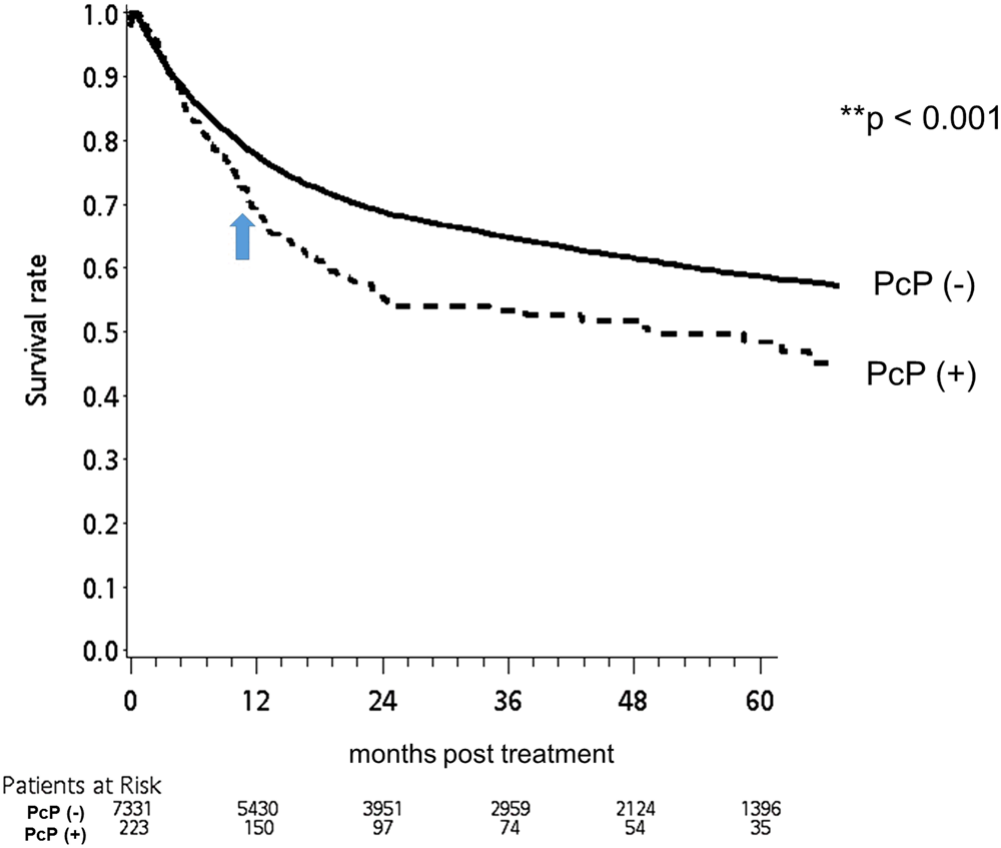
The Kaplan-Meier survival curve of the pneumocystis-infected (PcP(+)) and pneumocystis-free (PcP(−)) cases after chemotherapy (X axis: months post chemotherapy; Y axis: survival rate). Compared to pneumocystis-free patients, pneumocystis-infected patients had a significantly worse survival rate up to 9 months post chemotherapy.

**Figure 4:**
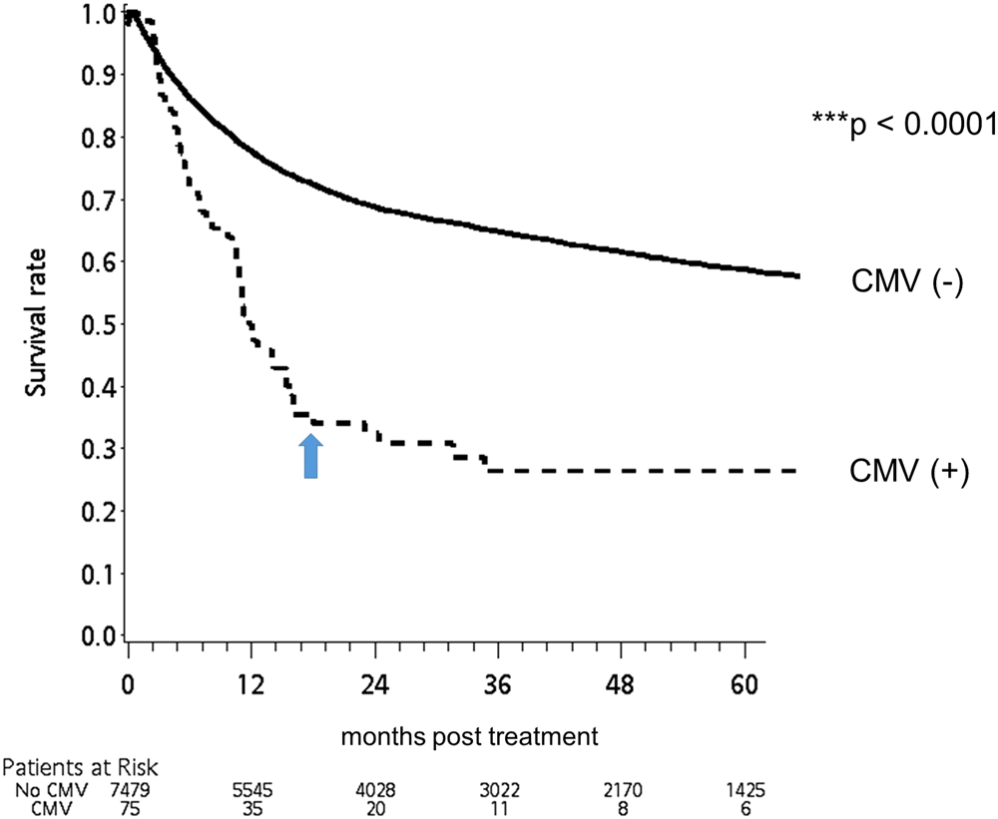
The Kaplan-Meier survival curve of the cytomegalovirus (CMV)-infected and CMV-free cases after chemotherapy (X axis: months post chemotherapy; Y axis: survival rate). Compared to CMV-free patients, CMV-infected patients had a significantly worse survival rate up to 18 months post chemotherapy.

**Figure 5:**
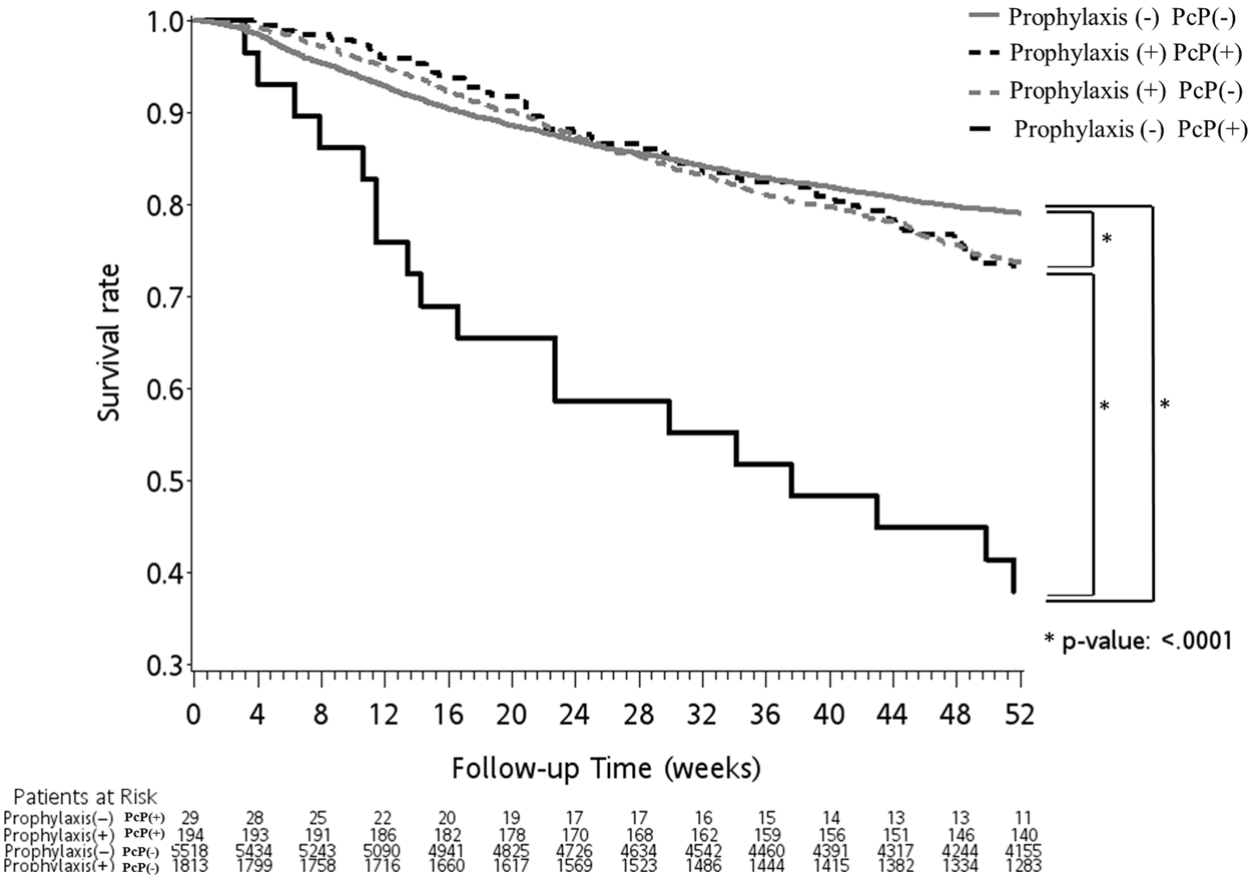
Pneumocystis prophylaxis has a beneficial effect on the overall first-year survival rate in HIV-uninfected, rituximab-treated non-Hodgkin lymphoma patients.

In the rituximab group, the all-cause mortality rates of the pneumocystis-infected patients at 30, 60 and 90 days post infection were 12.1%, 16.6% and 21.5%, respectively (supplement 2). Their survival rate was significantly inferior to that of the pneumocystis-free patients (Fig. 3). Notably, this detrimental effect persisted up to 9 months post infection (Fig. 3). A similar finding was found in CMV infection, another T cell-related opportunistic infection, which was associated with higher patient mortality up to 18 months post infection (Fig. 4). Further investigation showed that patients who contracted a pneumocystis infection without prior prophylaxis had the worst first-year survival rate, while the pneumocystis prophylaxis patients, whether or not they had a pneumocystis infection later in their treatment course, demonstrated a significantly better outcome (Fig. 5). Thus, simply by receiving pneumocystis prophylaxis with TMP/SMX, the overall first-year survival rate in this group of patients was markedly improved by 35% (38% vs. 73%).

## Discussion

Rituximab-based chemotherapy is one of the mainstream regimens for treating HIV-uninfected NHL patients because it offers better patient survival^18^. Results from both the national health insurance database and our hospital confirmed this finding (supplement 3). However, this better result comes at a price. The prevalence rate of pneumocystis infection is higher in patients receiving rituximab-based chemotherapy than in those receiving rituximab-free regimens^4,5,10^. Pneumocystis infection is an opportunistic infection that usually occurs when T cell function is suppressed. Thus, it is seen in diverse clinical entities, such as HIV infection, solid organ transplants, autoimmune disorders and chemotherapy patients^19–22^, as shown in our pilot study (supplement 4). To prevent this life-threatening complication, experts from various disciplines have published guidelines and recommended prophylaxis policies for these patients^15,16^. However, the necessity of pneumocystis prophylaxis in rituximab-treated, HIV-uninfected NHL patients is not yet certain. Therefore, we conducted this retrospective cohort study to see if pneumocystis prophylaxis with trimethoprim/sulfamethoxazole is beneficial to HIV-uninfected, rituximab-treated NHL patients. To assess this beneficial effect, we used first-year overall survival as the primary endpoint.

Our results revealed that the prevalence rate of pneumocystis infection in HIV-uninfected, rituximab-treated NHL patients was 2.95%, which was significantly higher than that of the control group. Similar results were reported by other researchers, including data from our centre (supplement 1)^4,5,10,23–25^. Once infected, these patients usually have a grave prognosis with a high mortality rate^1^. Moreover, due to its damaging effect on lung tissue, the pathological consequences can continue well beyond the active infection stage (Fig. 3). The role of comorbidities in the risk of getting pneumocystis infection is another important issue to clarify. Indeed, our results demonstrated that patients in the rituximab group had significantly more comorbidities, including chronic pulmonary disease, diabetes mellitus, chronic kidney disease and rheumatologic disease, than those in the control group. After careful adjustment with multivariate regression, neither age nor these comorbidities were shown to significantly increase the risk of pneumocystis infection. Furthermore, if the risk of pneumocystis infection is determined mainly by these comorbidities, other opportunistic infections should also increase, such as cytomegalovirus (CMV) infection. However, our study found that CMV infection, another T cell-related opportunistic infection, did not increase in both groups of patients (Table 1). This finding suggested that rituximab-treated patients had a unique tendency to acquire a pneumocystis infection. Therefore, in view of its serious outcome, pneumocystis infection in these patients deserves special attention, and prophylaxis should be considered, as in cases of other disease entities, such as solid organ or haematopoietic transplants^15^.

Pneumocystis infection can effectively be prevented by oral TMP/SMX, as shown by many researchers^15,16^. In the past, the main consideration against applying this prophylaxis measure in this group of patients was adverse drug effects related to TMP/SMX. However, most of the adverse events associated with TMP/SMX are reversible^26^, whereas the fatal outcome of pneumocystis infection is not. This assertion was well demonstrated in our study, in which we found that TMP/SMX prophylaxis provided a better overall first-year survival rate, while pneumocystis infection without prophylaxis led to a devastating outcome. It is worthwhile to note that there is currently no consensus to guide physicians in selecting which patients should have TMP/SMX prophylaxis^15^. Therefore, it is reasonable to assume that physicians would prescribe TMP/SMX prophylaxis mainly for those who have a higher disease grade or worse underlying conditions. It was against these odds that patients who had TMP/SMX prophylaxis still had a better overall first-year survival rate. Conversely, this result might be a reflection of the fact that timely diagnosing this fatal infection is difficult because its initial symptoms are nonspecific and there are few clinical characteristics guiding clinicians to identify patients at risk. This is also why a prophylaxis policy is suggested in many immunosuppressed patients^27^.

In a meta-analysis, Green *et al*. put together data from twelve publications and included 1245 patients^26^. With these data, they found that TMP/SMX prophylaxis effectively reduced the occurrence of pneumocystis infection by 91%, while 3.1% of the adult patients had to discontinue TMP/SMX due to drug-related adverse events, which were all reversible, according to the authors’ description^26^. Compared to Green’s meta-analysis, our study included more patients (N = 12, 158) and was more focused, i.e., it included HIV-uninfected NHL patients only. If we pooled all the HIV-uninfected NHL patients together, mixing the rituximab group and the control group, to mimic Green’s analysis, the prevalence rate of pneumocystis infection in these patients was 2.33% (284/12, 158). At the first glance, this result looks okay, but it is actually misleading because a high-risk group that we can save easily is masked. The fact is that the prevalence rate of pneumocystis infection was significantly higher in the rituximab group than in the control group (2.95% vs 1.32%), and thus, first-year all-cause mortality was higher. The prevalence rate of TMP/SMX adverse effects is dose dependent, except for the rare Stevens-Johnson syndrome. Importantly, unlike the pneumocystis prophylaxis policy of a decade ago, the current recommended dose of pneumocystis prophylaxis is less than half of the full treatment dose (TMP/SMX 400/80 mg once daily or 800/160 mg three times per week). Therefore, the prevalence rate of TMP/SMX adverse effects should be much lower than that in Green’s report. We therefore suggest that regular pneumocystis prophylaxis should be considered in HIV-uninfected, rituximab-treated non-Hodgkin lymphoma patients to improve their overall first-year survival.

There are several limitations of the current study. First, this study is not a prospective, randomized, double-blind study. The ICD-9 code cannot distinguish the exact stage of lymphoma and the general condition of each patient. Second, the patients in the rituximab group were older than those in the control group. The main reason for this difference is as follows: the patients in the rituximab group had B cell non-Hodgkin lymphoma, while those in the control group had B cell NHL or T cell NHL. In general, B cell NHL patients are older than T cell NHL patients. As a result, the rituximab group had a higher proportion of ageing-related underlying diseases, such as COPD, renal disease, and chronic kidney disease. Hence, in the initial analysis, there were differences between the two study arms in terms of age and comorbidities. However, a further multivariate analysis showed that only gender and rituximab impacted the risk of pneumocystis infection. Third, steroid treatment is a risk factor for pneumocystis infection. In our study, we were unable to collect the cumulative dose of steroid for each patient. That said, in current standard chemotherapy regimens for NHL, high-dose steroids are included in all the regimens. Therefore, most of the patients should have received a similar dose of steroid.

## Conclusion

HIV-uninfected NHL lymphoma patients had a significantly higher pneumocystis infection rate when they received rituximab-based chemotherapy. The patients who received pneumocystis prophylaxis had a significantly better overall first-year survival rate, whether or not they had a pneumocystis infection later in their treatment course. Considering the high mortality rate of pneumocystis infection and the availability of easy and effective prophylaxis measures, we recommend that pneumocystis prophylaxis be considered in rituximab-treated, HIV-uninfected NHL patients.

## Electronic supplementary material

Supplementary studies
