# Supplementary material for: Pneumocystis jirovecii pneumonia in HIV-uninfected, rituximab treated non-Hodgkin lymphoma patients
Source: Sci Rep. 2018 May 29;8:8321. doi: 10.1038/s41598-018-26743-4 (PMC5974272; doi:10.1038/s41598-018-26743-4)
Supplement: Supplementary file 1 — Supplementary studies [file 41598_2018_26743_MOESM1_ESM.pdf]

Title:

Pneumocystis jirovecii pneumonia in HIV-uninfected, rituximab treated non-Hodgkin lymphoma patients

Author lists: Kai-Che Wei MD [1], Chenglen Sy MD [2], Shang-Yin Wu MD [3], Tzu-Jung Chuang MS [4], Wei-Chun Huang MD PhD [4,5], Ping-Chin Lai MD PhD [6]

Supplement 1: Pilot study

Background: Although Taiwan National Health Insurance Research Database has larger scale and more patients, the detail of each patient in the database is hard to reaffirm. Complementary to these characteristics, medical records from a single center are easy to verify but have fewer patients. Therefore, we conducted this study in a single center to reconfirm our results from Taiwan National Health Insurance Research Database.

Aim: To investigate the beneficial effect of pneumocystis prophylaxis with trimethoprim-sulfamethoxazole in HIV-uninfected, non-Hodgkin lymphoma patients who had received rituximab-based chemotherapy in a single center.

Material and Methods: This is a retrospective study, which was approved by IRB in our hospital (IRB: VGHKS16-CT11-08). In this study, the medical records of HIV-uninfected, non-Hodgkin lymphoma (NHL) patients who had received at least three courses of rituximab-based therapies from January/1/2014 to September/30/2016 in Veterans General Hospital, Kaohsiung (VGHKS) were reviewed. To identify these patients, we included patients who had ICD-9-CM (the International Classification of Disease, 9th Revision, Clinical Modification) diagnosis codes for NHL (200.0–200.8 and 202.0–202.9). Their demographic data and diagnosis of pneumocystis infection were analyzed. Patients who fulfilled all the following criteria were defined as pneumocystis infection cases: 1. Positive *Pneumocystis jirovecii pneumonia* PCR (polymerase chain reaction) from either bronchial lavage fluid or sputum, 2. Image studies either chest X-ray or chest CT (computerized tomography) scan showed pneumocystis infection compatible changes, 3. Respond to anti- pneumocystis agents such as trimethoprim-

sulfamethoxazole (TMP/SMX). 4. Identified by codes for pneumocystis infection (136.3). Patients who were HIV infected, Hodgkin lymphoma or receiving hematopoietic stem cell transplantation were all excluded from this study

Results: In total, one hundred and three (N=103) HIV-uninfected, NHL patients were found during the study period in our hospital. Among them, three pneumocystis infection cases were identified. Two of them occurred in patients with diffuse large B cell lymphoma, while the other case was a patient having follicular lymphoma. Thus, the prevalence rate of pneumocystis infection among HIV-uninfected, rituximab treated NHL cases was 2.91% (3/103) in VGHKS. (Supplement table 1)

Type*	Number	Pneumocystis infection
Diffuse large B cell lymphoma	87	2
Follicular lymphoma	9	1
Other types of lymphoma **	7	0
Total	103	3 (2.91%)

Supplement table 1. The prevalence rate of pneumocystis infection in HIV-uninfected, rituximab treated non-Hodgkin lymphoma patients in a medical center in Taiwan.

From 01/01/2014 to 30/09/2016, the prevalence rate of pneumocystis infection among HIV-uninfected, rituximab treated NHL patients (N=103) was 2.91% in VGHKS. All patients included in this study had received at least three courses of rituximab-based chemotherapies.

*: Those who had received autologous hematopoietic stem cell transplant were excluded.

** Other types of lymphoma included mantle cell lymphoma, intravascular large B cell lymphoma, and mucosa associated lymphoid tissue lymphoma (MALToma) with large cell transformation.

Supplement 2. The survival rate following pneumocystis infection in rituximab group

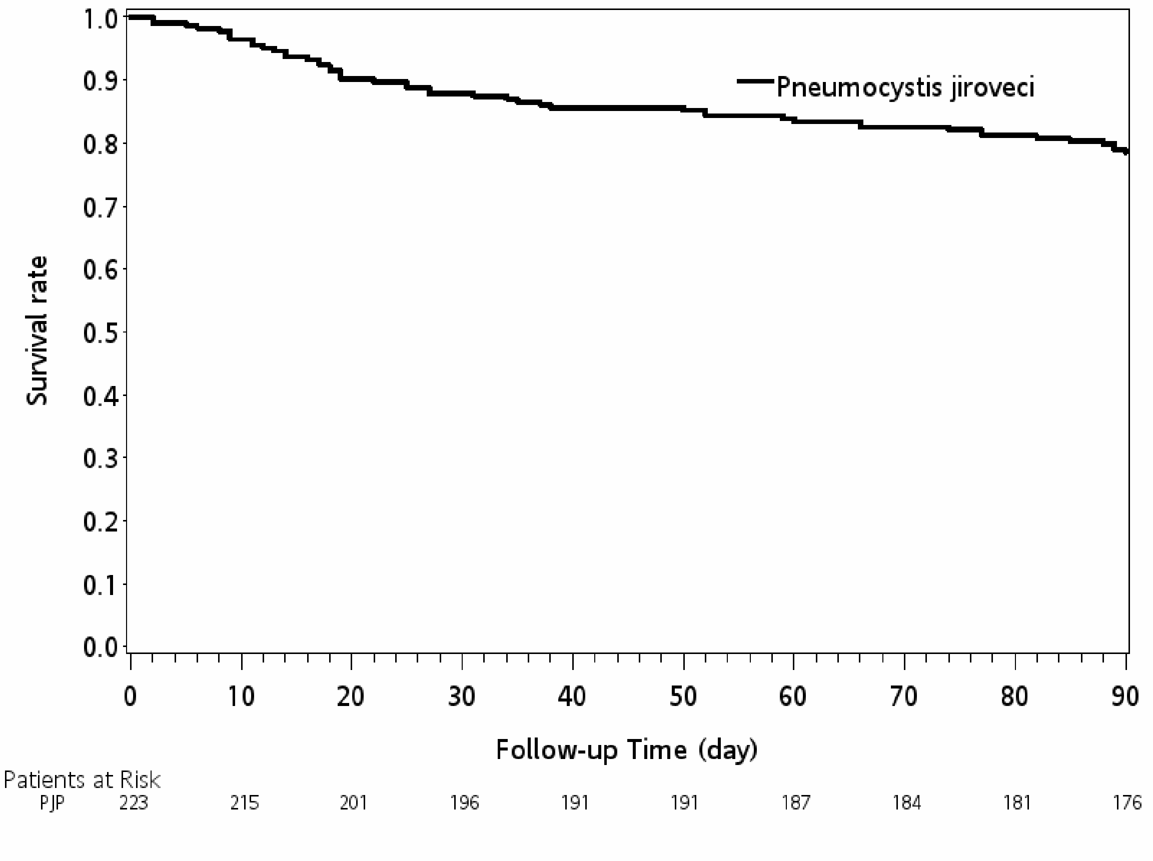

Supplement figure 1. The short term survival rate following pneumocystis infection in rituximab group

In this study, we included newly diagnosed HIV-uninfected, non-Hodgkin lymphoma patients in HV database & National Health Insurance Research Database from 2006/01~2013/12 (ICD 9: 200, 202). Details of inclusion/exclusion criteria are listed in the main text material and method section. Among 7554 patients who received rituximab-based chemotherapy, two hundred and twenty three patients (N=223) had pneumocystis infection. The results showed that the all-cause mortality rates of pneumocystis infection cases at 30, 60 and 90 days post infection were 12.1%, 16.6% and 21.5%, respectively.

Supplement 3: Pilot study

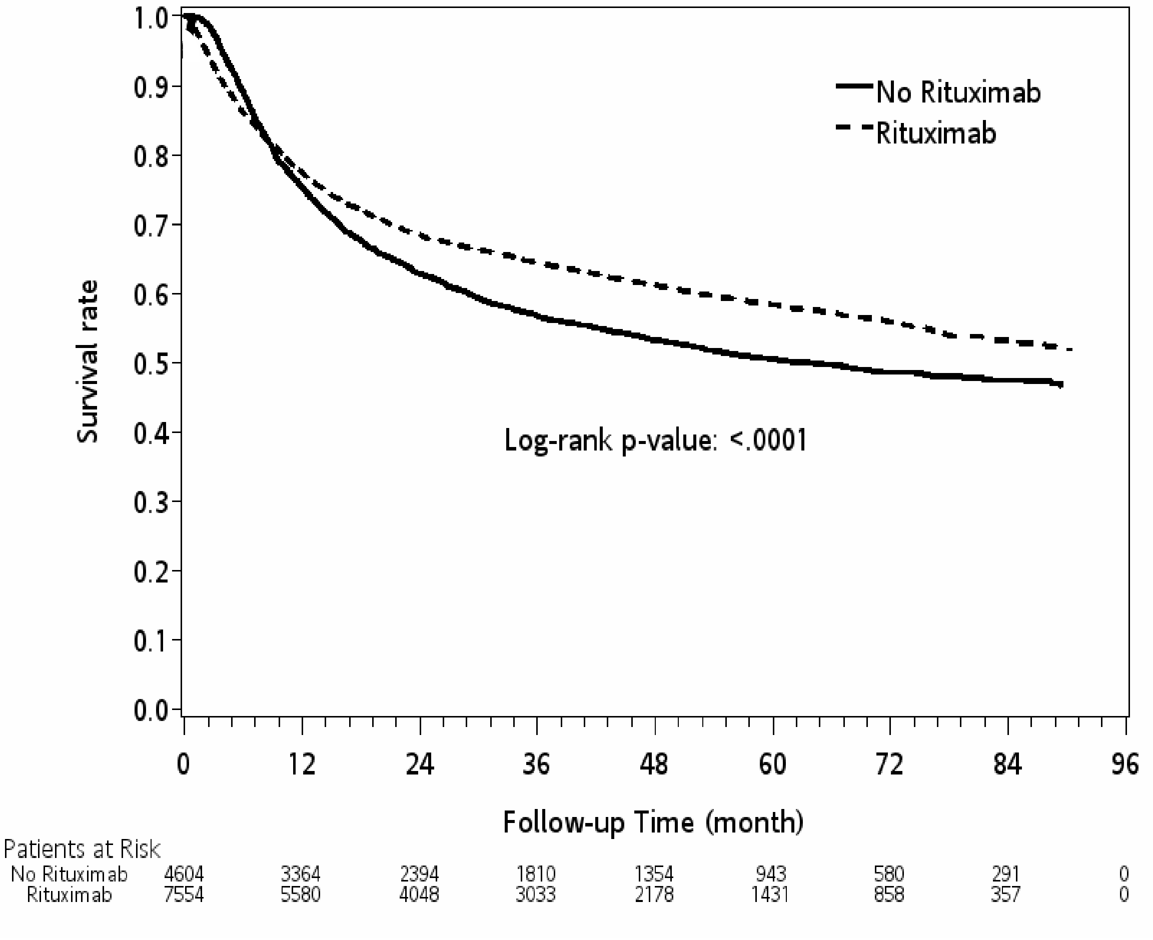

Supplement figure 2. The survival rate of rituximab-based vs. rituximab-free chemotherapy in HIV-uninfected non-Hodgkin lymphoma patients

In this study, we included newly diagnosed HIV-uninfected, non-Hodgkin lymphoma patients in HV database & National Health Insurance Research Database from 2006/01~2013/12 (ICD 9: 200, 202). Details of inclusion/exclusion criteria are list in the main text material and method section. The results showed that rituximab-based chemotherapy offers better patient survival than rituximab free regimen. (rituximab group N=7554, rituximab free group N=4604)

Supplement 4. Pilot study

Pneumocystis infection	N (%)
HIV/AIDS without lymphoma	22 (50%)
HIV/AIDS with DLBCL &rituximab	2 (4.5%)
Non-HIV with rituximab*	5 (11.4%)
Others without rituximab	15 (34.1%)
Total	44 (100%)

Supplement table 2. During 2014.05~2016.09 in VGHKS, pneumocystis infection were diagnosed in 44 patients who had various underlying diseases

DLBCL = diffuse large B-cell lymphoma

*: These included three cases of lymphoma, one case of pemphigus vulgaris and one case of idiopathic thrombocytopenic purpura.
